# Maximising engagement and participation of intellectual disability staff in research: Insights from conducting a UK-wide survey

**DOI:** 10.1177/1744629520924141

**Published:** 2020-05-12

**Authors:** Claire Kar Kei Lam, Jane Bernal, Janet Finlayson, Stuart Todd, Laurence Taggart, Annette Boaz, Irene Tuffrey-Wijne

**Affiliations:** Kingston University and St George’s, University of London, UK; University of South Wales, UK; Glasgow Caledonian University, UK; University of South Wales, UK; University of Ulster, UK; Kingston University and St George’s, University of London, UK; Kingston University and St George’s, University of London, UK

**Keywords:** difficult topic, intellectual disability staff, research methods, response rates, surveys

## Abstract

**Aim:**

This article explores ways of maximising engagement of intellectual disability staff as research participants, research advisers and research implementers.

**Method:**

The authors describe and reflect on a three-phased strategy in recruiting front-line staff (*n* = 690) working for intellectual disability service providers (*n* = 25) to participate in a UK-wide anonymous online survey about death, dying and bereavement.

**Results:**

Important elements in engaging participants were: involving stakeholders at all stages of the research process, which includes: building relationships with participating organisations; enlisting organisational management support at all levels; an attractive and well laid-out collection tool; a well-structured recruitment strategy; time and flexibility; and a varied and targeted dissemination strategy. However, the recruitment method had limitations, in particular around representativeness, bias and generalisability.

**Conclusions:**

Staff in intellectual disability services can be enthusiastic and invaluable research participants. Active engagement between researchers, participating organisations and stakeholder groups is key to ensuring involvement of intellectual disability staff with research.

## Background

This article focuses on ways of maximising research engagement of staff working in intellectual disabilities services, mostly in terms of recruiting intellectual disability staff as research participants, but also in terms of their contributions as research advisers and research implementers. Active engagement of staff with research is one way of bridging the gap between research and practice. This, in turn, can facilitate changes in staff attitudes and in the way things are done, promote improved health and well-being of staff and clients and influence policy changes (Read, 2018). Achieving this can be especially challenging when the research is concerned with a difficult, or even a taboo topic; we draw on our experience of doing research around dying, death and intellectual disability.

In particular, this article reflects on the methods, processes and outcomes of a UK-wide survey of 690 staff working with people with intellectual disabilities in residential and supported living services, and how these research processes affected stakeholder engagement with the study. The *Talking About Dying Survey* (TADS) investigated how the staff communicate about death and dying with people with intellectual disabilities who are dying or bereaved; how they confront issues of death, dying and bereavement at work; and how often they were confronted with such issues. The survey was developed and conducted in 2017–2018 by the authors, a team of collaborating researchers based at four universities across all four UK countries (Tuffrey-Wijne et al., 2020).

There have been many studies where intellectual disability staff took part as research participants and informants. For example, with regard to our own research topic, a systematic literature review on the experiences of staff who support people with intellectual disabilities around death, dying and bereavement found 13 papers reporting studies where front-line care staff and/or managers were the participants (Lord et al., 2017). Eleven of these were qualitative studies involving focus groups or interviews, one was a qualitative questionnaire (*n* = 38) (Forrester-Jones, 2013) and one was a survey with qualitative responses (*n* = 57) (Hoover et al., 2005). These staff-focused studies highlight the importance of investigating staff perceptions, knowledge and attitudes, as appropriate support for people with intellectual disabilities around death and dying depends on staff who feel confident and are well supported themselves.

A Dutch survey of staff working directly with people with intellectual disabilities who have end of life care needs (Bekkema et al., 2015) included 294 questionnaires sent to care staff employed by care services for people with intellectual disabilities; of these, 196 (67%) were returned. These staff were more highly educated than most front-line intellectual disability support staff in the United Kingdom: 85 were registered nurses, 8 were certified nursing assistants and 103 were social workers; all were members of a national research panel, which may explain the good response rate.

Hunt et al. (2019) report on a survey investigating end of life care outcomes for people with intellectual disabilities living in residential care in the United Kingdom. They sent a detailed post-bereavement questionnaire to 188 intellectual disabilities staff involved in the support of a person with intellectual disability who was known to have died, of which 158 were returned. Core details about 222 deaths, and the staff contact details for 188 of these, had been obtained from the intellectual disability service providers.

There is a dearth of literature around the methodological issues of involving intellectual disability staff as research participants and informants. The published papers indicate that at least some intellectual disability staff are willing to participate in research on difficult topics, but they do not provide much detail on the design and conduct of death-related studies within intellectual disability services; nor do they shine a light on how to engage staff with the topic. There is little evidence detailing the process of recruiting care staff, outlining any pitfalls when involving support staff in intellectual disability research, or outlining recommendations for successful engagement and satisfactory levels of participant recruitment.

Hall et al. (2017) conducted a small focus group study of intellectual disability staff, aimed at identifying ways to maximise recruitment of study participants with intellectual disabilities, facilitated by intellectual disability staff. They made three suggestions for researchers, which may also be of relevance for studies where the staff themselves are the main participants: (1) flexible contact methods, aided by making use of digital avenues; (2) forming stronger relationships between universities and care companies, which can help disseminate research findings, and develop continued collaborations in future studies; and (3) approaching managers as the main contact, as supportive managers are the key factor to staff recruitment. This confirms a suggestion by Tuffrey-Wijne et al. (2008) that intellectual disability staff are more likely to participate (or facilitate participation of people with intellectual disabilities) if they can see personal benefit, such as useful study outcomes or the cathartic effect for the study participant of engaging with researchers on a sensitive topic.

Our objectives for this article are to describe our strategy in recruiting intellectual disability support staff to participate in our survey, and to explore and evaluate the factors that affected the engagement between researchers and practitioners, including the response rate and the engagement of intellectual disability managers and front-line staff with the disseminated findings. We will also explore the limitations to our methods, some of which were significant. We hope this will be of use to anyone planning to conduct research involving intellectual disability staff and services, not just in the United Kingdom, but internationally.

## Methods

### The *Talking About Dying Survey*

The TADS questions were based on the research evidence of current issues and best practice with regard to death-related communication (Northway et al., 2018; Ryan et al., 2011; Stancliffe et al., 2016; Todd, 2013; Tuffrey-Wijne, 2013; Tuffrey-Wijne and Rose, 2017; Wiese et al., 2013). The study objectives included gauging how current practice relates to this evidence base, as well as adding to the evidence by gathering new data on staff issues and concerns. This, in turn, would inform the development of guidance and training. We received 690 completed online questionnaires from staff working for intellectual disability providers of residential and supported living services across the United Kingdom (61% response rate). Of these respondents, 68% were direct support staff, 29% were front-line managers and 3% were ‘other’. The study findings are reported elsewhere (Tuffrey-Wijne et al., 2020).

### Research Advisory Group

The Research Advisory Group (RAG) comprised 16 members lending different expertise and perspectives to the TADS research team, including 3 people with intellectual disabilities, 2 family carers of people with intellectual disabilities who had been bereaved or had died, 2 senior managers from intellectual disability service providers, 1 intellectual disability nurse, 2 intellectual disability support staff and 4 academic researchers. Several of these stakeholders were involved from the time of developing the study protocol. The RAG met four times throughout the project, from before the design of the questionnaire through to the dissemination of findings towards the end of the project. This stakeholder involvement ensured that the research questions and study design were relevant to its target audience. For example, a family carer pointed out the importance of asking questions about people’s understanding of death. Her adult son with Down syndrome knew that his father had died, but had difficulty understanding that he would never come back, making it more difficult for the mother to talk about the death with her son. As a result, we included two questions related to the person’s understanding of the universality and permanence of death (‘everybody dies’ and ‘people who die can never come back’). Senior managers from intellectual disability service providers held additional conference calls with the research team to discuss participant recruitment strategies, including the feasibility for their staff to complete a 20–40 min survey online.

### Survey design

This was an anonymous questionnaire, using an online survey tool (SurveyMonkey©). Staff who had experience of supporting a person with intellectual disabilities in their care who had died, was terminally ill or had been bereaved in the past year, were asked detailed questions about this person and about their own experience of supporting them. On a topic as difficult as death and dying, great sensitivity was needed. It was essential that the survey was well presented and engaging, and that respondents felt it was relevant to them. All RAG members helped trial the survey by completing it themselves and sharing it with peers; they then met together to share feedback. This enabled us to assess how long it took to complete the survey and where the risk of respondents abandoning the survey was the greatest. At these risk points, we inserted photographs and graphics, for example, a photo of two research advisers with intellectual disabilities holding up encouraging signs saying *Please keep going! It will really help to know more about this.* Edits were made to clarify, rephrase and replace unnecessary or unclear questions. This was helped by using the software’s internal logic tool, which ensured that questions that were not relevant to a respondent were skipped automatically. We ensured that respondents’ answers would be saved automatically as long as they returned to the same device and browser; this allowed respondents to quit and return to the survey at any time, which was useful as staff often had several other demands on their time. The survey was trialled in all available formats (tablets, mobile phones and desktop computers) with 39 stakeholders and adjusted in light of their feedback, before inviting participants.

### Three-phase participant recruitment

**Figure 1.**
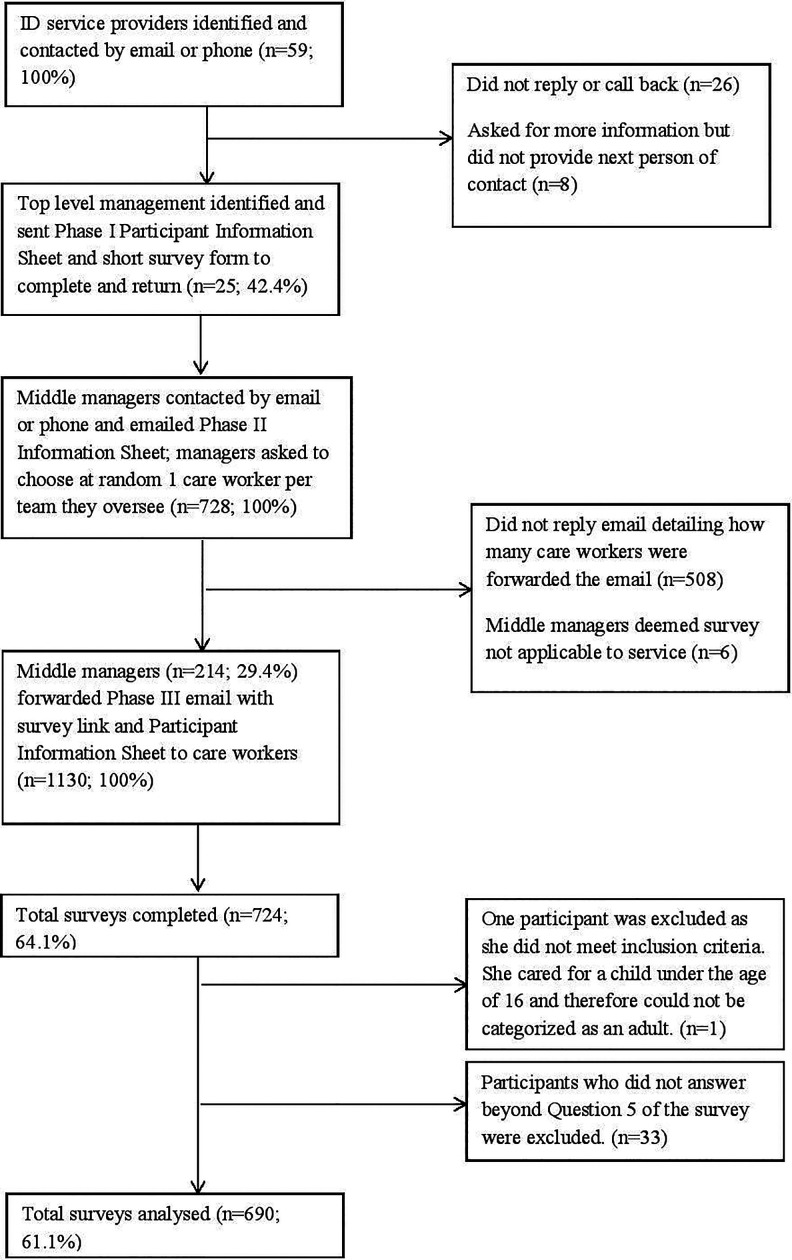
Recruitment to the *Talking About Dying Survey*.

The targeted survey respondents were support staff working directly with people with intellectual disabilities, either with or without recent experience of death or bereavement among the people they supported. We utilised a top-down recruitment approach in three separate phases: (I) recruiting organisations, (II) recruiting middle managers and (III) recruiting survey respondents (direct care staff) (see Figure 1).

We decided that only one staff member per team or residence should be invited to complete the survey. This was not only to ensure that the burden on staff teams was not too great, but also that there would not be more than one survey response relating to a particular deceased or bereaved person with intellectual disabilities, as this would skew the findings in relation to the profiles of people with intellectual disabilities who have died or are bereaved. The survey was anonymous, partly to encourage honest responses, and partly to comply with the demands of the Faculty Research Ethics Committee (REC) of Kingston and St George’s University (London), which approved the study. The REC prevented us from contacting any respondents directly, in contrast to the survey by Hunt et al. (2019), where intellectual disability staff who had supported a deceased person with intellectual disabilities were identified by the research team and non-responders could be followed up. Our gatekeepers were ‘middle managers’, that is, those with direct managerial responsibility for a range of staff teams, whom we relied on to select potential respondents, help us relay information to their staff such as the survey link, reminders and information sheets to the intended participants.

The anonymous nature of the survey probably helped respondents to give honest responses, but it also had drawbacks. We had no way of identifying (e.g. by initials, date of birth or location) the people with intellectual disabilities whose death or bereavements staff were describing. The approving REC ruled that ethical considerations also prevented us from allocating a unique identifying number to each care setting, because it might have enabled either the staff member or the person with intellectual disabilities to be identified. There was, therefore, no way to detect when more than one staff member from a single setting had sent in a response, other than the original direction to middle managers to send the survey to only a single member of support staff in each setting. We have some evidence of one likely instance of double counting, where several respondents described a person with intellectual disabilities who had died of an extremely rare condition, with similar other characteristics, in the same county. The inability to detect double counting was a potential flaw in this methodology. It negatively affects our confidence in some of the findings, in particular those findings relating to the profiles of deceased or bereaved people with intellectual disabilities.

#### Phase I: Recruiting organisations

A total of 59 intellectual disability service providers within the United Kingdom were identified from the research team’s extensive networks and contacts. Additional contacts were made during meetings at conferences, as well as from internet searches upon suggestions from the RAG. They provided a range of services for people with intellectual disabilities including supported living, residential care and outreach support, and varied in size and reach, with the smallest organisations supporting under 50 people with intellectual disabilities within one local area and the largest supporting over 3000 people across the United Kingdom. The most senior organisational manager was identified and contacted by email. In total, 25 services (42%) agreed to take part via written consent from a top level manager, who then provided us with the contact details of their middle managers. While these 25 services represented a large sample, the phase I recruitment method introduced selection bias, with organisations known to the researchers more likely to be invited, and organisations where the senior manager had a positive attitude towards ‘talking about dying’ probably more likely to take part.

#### Phase II: Recruiting middle managers

The 25 senior service managers provided the contacts of a total of 728 middle managers. We contacted all of these with the invitation to send the survey link to their staff; a total of 214 middle managers responded, and between them reported that they had sent the survey link to 1130 staff members. Phase II presented us with a number of challenges, including the strong possibility of response bias, with managers probably more likely to select staff who had a particular experience of death or bereavement. It also affected our ability to get an accurate picture of the response rate, as we relied on these middle managers to let us know how many surveys they decided to send out.

This phase therefore required the most time, as it was crucial to the success of the study that middle managers sent the link to the right staff and encouraged these staff to participate. At their request, preview link was created especially for managers, allowing them to look at the survey in its entirety without answering any survey questions. This helped to encourage them to promote the survey to their staff. With regard to the contact method, we had planned to make initial contact by telephone, to be followed up by emails. However, it soon became apparent that emails were the preferred mode of communication. Many service managers were out in the field and hard to reach over the telephone; on occasions where contact was successfully made over the phone, managers were often engaged with other priorities and asked to be sent more detailed information by email. Emails therefore became the standard initial mode of contact. A follow-up telephone call was offered where managers preferred, but of the 214 managers who responded to our phase II recruitment, fewer than 5 asked for a telephone call.

#### Phase III: Recruiting survey respondents (direct care staff)

A total of 724 completed the survey over a 6-month period; after excluding those who did not answer any questions beyond the first few, 690 useful responses remained. If the number of 1130 care staff who were reportedly sent the TADS link is correct, this represents a 61% response rate. On average, participants spent about 10 min completing the survey online; a further 20–30 min was spent by participants with the experience of caring for someone who had died, was terminally ill or had been bereaved. Results show that staff were willing to engage and had many opinions to share. Having managers who were supportive of the study probably helped with this.

Beyond motivating phase I and phase II managers, efforts to engage front-line staff were perhaps the most challenging yet most crucial, as they were ultimately our survey respondents. Since our recruitment at this stage relied on indirect contact, it was important to send a clear and engaging email that middle managers could forward to their staff at the click of a button. We believe this, in combination with a well-presented survey, was effective in engaging and motivating staff to respond.

### Elements of successful engagement of intellectual disability services and staff in research

**Figure 2.**
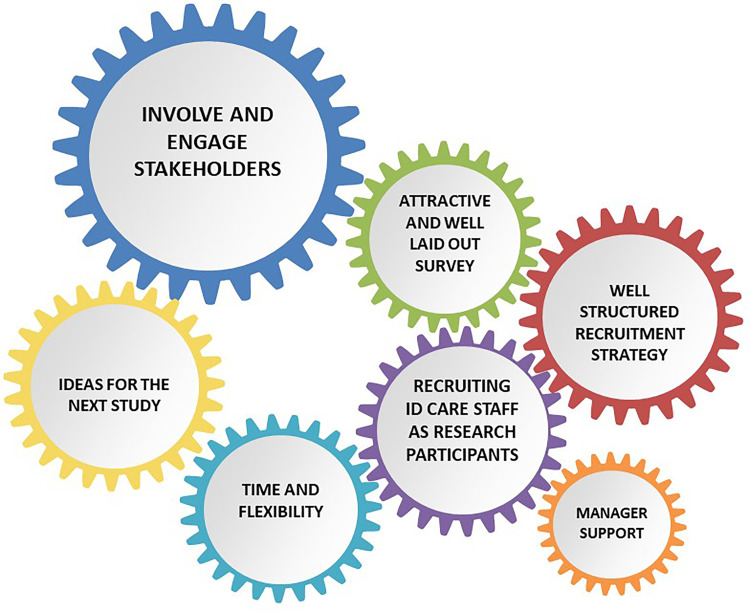
Elements of successful engagement of intellectual disability services and staff in research.

We found a number of interlinked strategies to be helpful for engaging intellectual disabilities staff at different stages of our study (see Figure 2). The following will describe these in further detail.

#### Involve stakeholders at all stages of the research process

There was a significant networking advantage to having the RAG members involved throughout the project. This helped not only with identifying and recruiting organisations, but also to develop a realistic timeline and help the research team understand how potential participants could be approached in different types of intellectual disability services. The RAG also helped to ensure that the survey questions were of high relevance to service providers, participants and those who would be interested in our findings. The importance of high-level organisational engagement was demonstrated by the fact that the two services from our RAG eventually accounted for about half of all surveys sent out (based on self-reports from middle managers – the research team was unable to trace the anonymous responses back to particular organisations). This was due partly to their large geographic spread and scale of their services, but also, undoubtedly, to management support for the project. The RAG members became advocates for the study within their organisations. The drawback of such support and engagement of specific services is the real possibility of response bias, as we discussed earlier.

Stakeholder engagement within the RAG was fostered by the involvement of people with intellectual disabilities, who required us to be creative in the way meetings were run, and needed everyone to be clear in their communication and clear about their role within the project.

At the data analysis stage, the research team presented the RAG with several possible directions for data analysis of over 200 questions from our survey. The RAG also helped to decide what kind of dissemination materials and methods would be useful to their own stakeholder groups, resulting in a range of different outputs. The RAG members were involved in developing and disseminating these materials.

Stakeholder engagement went beyond the collaboration with the RAG. Building a relationship between the research team and the participating organisations meant that there was ongoing organisational commitment to the study. This needs to be a reciprocal relationship, with the participants benefiting from the study, even if it is by understanding that their contributions will help others. The ‘giving back’ by the research team can take time, as the period between recruitment, data collection, data analysis and dissemination can be lengthy, especially in a phased study. Some of our phase I contacts were made over a year before the final analysis. The researchers remained in occasional email contact with the phase II managers, until they were able to send the final report.

#### Enlist management support at all levels

The support and engagement of managers at phases I and II was crucial. At the most senior level, the most successful recruitment was in organisations where face-to-face contact was made with a manager who was able to discuss and promote the study within the organisation. Some took a long time over this, waiting for the right meetings and including a piece about the study in their nationwide internal newsletter (which we had written for them at their request). It paid off, as more responses came in after the middle managers in these organisations started sending out survey links to their staff. Key to management engagement was ensuring that all those involved felt that participating in the TADS was relevant and worthwhile to their organisation and beneficial to their staff. In phase II, much time was spent communicating with middle managers to ensure that they felt that each staff member’s participation really mattered.

We found that emails to middle managers were more likely to get a response if they were personalised, for example, by using the managers’ names several times throughout. We also included a pre-set response email which could be quickly completed with details such as the number of staff they had forwarded the survey link to; managers reported that they liked the fact that a return email was already structured for them. Additionally, providing a short and succinct follow-up email after 2 weeks, with a reminder of the time frame of the study, was welcomed by middle managers who were yet to respond. Although personalising emails greatly increased the amount of researcher effort and time, it appeared to be worth it, as response rates increased. We hypothesised that the rise in responses was due to an increased sense personal responsibility and recognition of their key role in research. On the downside, this approach may also have led to over-recruitment (e.g. enthusiastic managers inviting more than one staff member per home or team), but we had no way of checking this.

#### Attractive and well laid-out data collection tools

As explained above, it was crucially important to have a well-presented and attractive looking survey, making full use of technology. Free text feedback on the survey itself was mostly positive (‘Very good survey, thank you!’; ‘Nice survey’). A few respondents reported that they found the topic upsetting (‘I found this really hard to fill out as my emotions took over and it felt as though everything I had dealt with came back and re-surfaced’). We received over 1500 optional free text responses, where respondents explained their answers in further detail, sometimes at length; this suggests positive participant engagement with the survey.

#### Well-structured recruitment strategy

In developing the recruitment strategy, it is important to consider carefully who the key gatekeepers are, and who will be able to be a champion of the study within the organisation. In our study, having a three-phase recruitment strategy was time-consuming, but it was time well spent. Our strategy required significant effort and planning, with implications for budgeting and the use of researcher time.

#### Time and flexibility

It is important to note that we allocated 6 months for recruitment and data collection, allowing enough time for information to pass on from senior management to front-line staff members. While the three-phased, top-down design of our recruitment had obvious benefits and ensured participation of the entire service, recruitment efforts were in effect tripled due to the involvement of higher managers, middle managers, as well as support workers. A 6-month period not only provides flexibility for middle managers to respond, but also demonstrates respect to the varying schedules and demands of both managers and their staff, accounting for annual leaves, competing work priorities; and allows time for further clarifications when needed.

Flexibility was also important. We adjusted our strategy throughout, in response to manager and participant feedback. Giving participants the flexibility to complete the survey in their own time, with the help of technology, was also useful.

#### Targeted and varied dissemination

Dissemination activities were developed together with the RAG members, who helped the research team to understand what kind of outputs or activities would be useful to their stakeholder groups. The data were analysed with support and input from the RAG. There was a vast amount of data, and we wanted to be sure we focused on the aspects that would be most useful to stakeholders. For example, with regard to survey responses to questions around ‘Talking about dying with people who are facing bereavement’, our advisers with intellectual disabilities wanted to know more about the extent to which staff talked to people with intellectual disabilities about this; one adviser commented, ‘No-one sat down with me to talk about stuff; I had an idea she [relative] was dying, but not properly’. Service managers wanted to know more about staff support: ‘It will be good to know what staff felt would help them’. Subsequently, in dissemination activities, these were some of the elements we focused on. A free-of-charge 1-day feedback conference was held in London, where staff working for intellectual disability service providers who had participated in the study were given priority to attend. All 728 managers from phase II were sent a summary of the survey findings, along with quotes from respondents and top tips (Tuffrey-Wijne, 2019b). In addition, family carers, people with intellectual disabilities and a senior manager from the RAG helped to produce short videos related to the TADS (Tuffrey-Wijne, 2018a, 2018b, 2019a). A Twitter masterclass was held over 5 days, followed by around 8000 people. For example, as a result of the finding that most people with intellectual disabilities attend the funeral of a loved one, but very few are actively involved in it, one of the Twitter threads was around involving people with intellectual disabilities in funerals. Twitter comments included ‘Thank you – I’ve never even considered this’, and ‘We’ve seen it so many times “it’s not in a person’s best interest” to go along to a funeral, let alone be involved’.

As a result of the survey and its outcome, several intellectual disability service providers reported that they had initiated staff training on death, dying and bereavement. The process of engagement between researchers and stakeholders also helped with the identification of future areas for research.

## Discussion

Involving intellectual disability staff as research participants not only helps to understand more about the experiences of people with intellectual disabilities whose own involvement as research participants may be difficult (proxy informants), but also the perspectives and practices of staff themselves.

Good levels of research participation are important. Although our recruitment efforts required a substantial amount of time and effort, it was beneficial as we achieved a good response rate of 61%, in line with what is considered the target rate (60%) (Fincham, 2008), and a higher than what is expected of a web survey (11% lower than non-web survey modes) (Fan and Yan, 2010).

The elements of success in recruiting a sufficiently large sample and engaging the participants rested heavily on the engagement of intellectual disability services throughout the study. This was therefore not restricted to the period after the study design stage and before the closing of data collection period, but rather, an ongoing feature at every stage of the study. Ensuring reciprocity, by providing feedback from researchers to participants, is important (Lewis and Porter, 2004; Phillipson et al., 2012).

Hall et al. (2017) found supportive managers to be the key to successful recruitment, reporting on the difficulty for staff to engage with research when unsupported by senior management. They found that senior managers’ perception on the importance of research greatly determined how much time and resource were allocated, subsequently affecting whether or not staff would be able to participate at all in research. Many of our elements of success outlined above echo these findings.

Our study expands on the suggestions by Hall et al. (2017), highlighting in addition the importance of early stakeholder involvement and networking, the importance of a well-presented data collection tool, and a well-structured recruitment method. For researchers, the emphasis on fostering a RAG leading to a better designed survey and recruitment procedure not only increases respondent rates, but supports dissemination that is relevant to stakeholders. Ultimately, having the ability to transfer knowledge from the outcomes of a study to key interest groups increases the value of its findings and research efforts. For participants, there are obvious benefits of learning about results and translating the implications for practice. In our study, this spreads from the organisation level down to the individual choices each care worker makes. On the other side of the same coin, participants also affect change by informing research, fostering a greater sense of personal agency towards improved standards of practice. The increased engagement with research at all levels and in multiple directions promotes greater recognition of care organisations and staff, evidenced-based practice, and helps bridge the gap between theory and practice.

### Limitations

All research methods and approaches to participant recruitment have their limitations, which must be considered in relation to the study’s aims and objectives. There were a number of limitations within our study design and recruitment procedures. Our initial research aims included an investigation of the proportion of people within intellectual disability residential and supported living services who are diagnosed with an irreversible terminal condition, or are bereaved, within a 12-month period. Because of the anonymous nature of the study and the issue of possible double counting, we could not meet this aim. Furthermore, the reliance on gatekeepers (in our study, these were the ‘middle managers’) to select participants led to response bias and some uncertainty about precise response rates. It is important to anticipate and be transparent about such limitations, and to find ways to mitigate them if at all possible. Our participant recruitment method was suitable for investigating staff experiences and perspectives, but not for establishing prevalence of death, dying and bereavement within intellectual disability services. Future researchers who are interested in prevalence rates of particular issues or person characteristics would need to find a way that rules out double counting without compromising confidentiality.

The focus of this article is on the extent to which our methods were effective in recruiting and engaging participants. In this, we were limited by the fact that we did not formally evaluate this. Given the importance of ‘pathways to impact’, it would be useful in future studies to incorporate a proper evaluation of the impact of various approaches to participant recruitment and dissemination strategies.

However, our analysis of what helped to improve engagement (including recruitment rates) is, we believe, transferable to other studies and other settings, both within the United Kingdom and internationally. In particular, we would advocate stakeholder involvement and ongoing active engagement between research teams and participating organisations, at all stages of research, regardless of the study methods.

## Conclusion

Involving and engaging stakeholders throughout a research project is an important aspect of the research impact pathway. For research in intellectual disability services, these stakeholders include people with intellectual disabilities, family cares, service managers and front-line intellectual disability staff. Their involvement helps to ensure that the right questions are asked; data collection tools are well presented; recruitment procedures are adequate and well-structured; and, as a consequence, recruitment rates are maximised. It also helps to target dissemination activities, thus ensuring maximum research impact. Time and flexibility is required, which has resource funding implications.

In our study, intellectual disability staff were invaluable and enthusiastic advisers and participants, who were able to provide unique insight into best practice for talking about loss with people with intellectual disabilities. Bridging the research–practice gap in the field of intellectual disability will ultimately lead to improved support for people with intellectual disabilities. Front-line intellectual disabilities staff can play a crucial role in the bridging of this gap.
